# A Portable Lipid Bilayer System for Environmental Sensing with a Transmembrane Protein

**DOI:** 10.1371/journal.pone.0102427

**Published:** 2014-07-29

**Authors:** Ryuji Kawano, Yutaro Tsuji, Koki Kamiya, Taiga Kodama, Toshihisa Osaki, Norihisa Miki, Shoji Takeuchi

**Affiliations:** 1 Artificial Cell Membrane Systems Group, Kanagawa Academy of Science and Technology (KAST), Takatsu-ku, Kawasaki, Japan; 2 Department of Mechanical Engineering, Keio University, Kohoku-ku, Yokohama, Japan; 3 CIRMM-IIS, The University of Tokyo, Meguro-ku, Tokyo, Japan; Texas A&M University, United States of America

## Abstract

This paper describes a portable measurement system for current signals of an ion channel that is composed of a planar lipid bilayer. A stable and reproducible lipid bilayer is formed in outdoor environments by using a droplet contact method with a micropipette. Using this system, we demonstrated that the single-channel recording of a transmembrane protein (alpha-hemolysin) was achieved in the field at a high-altitude (∼3623 m). This system would be broadly applicable for obtaining environmental measurements using membrane proteins as a highly sensitive sensor.

## Introduction

Planar bilayer lipid membranes (BLMs) have been proposed as a useful platform for various potential applications including single ion channel analysis, drug screening, nanopore sensing at the single-molecule level, and nanopore DNA sequencing. [1]–[3] For sensing with a BLM system in outdoor environments the following properties are imperative: 1) portabilization of the measurement system, 2) low noise measurement, and 3) preparation of reproducible and stable BLM. Although several types of lipid bilayer micro-chips are reported, [4]–[7] none of the systems have been satisfied with these requirements at the same time.

**Figure 1:**
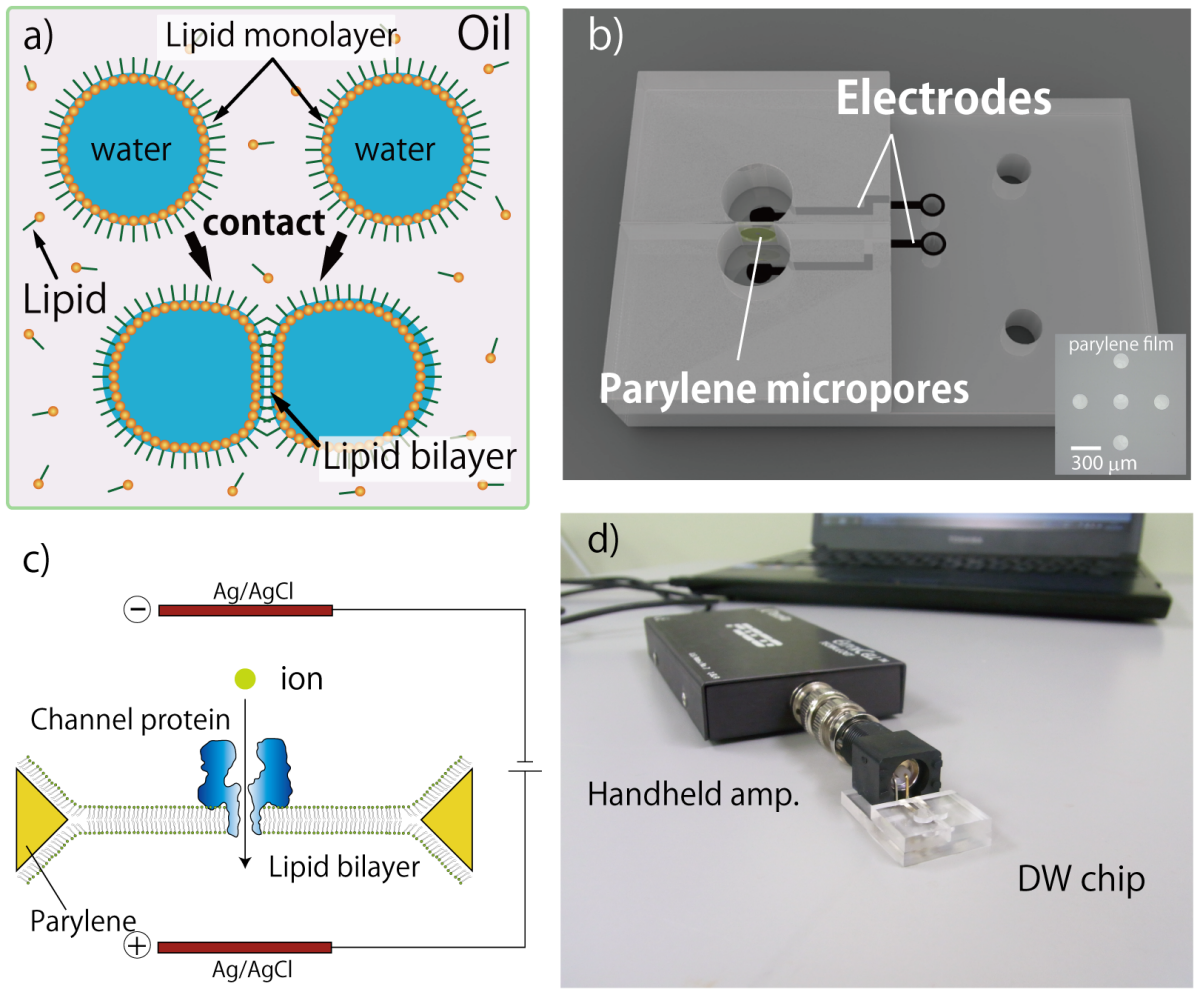
Portable system for ion channel current recordings. Droplet contact method for reproducible and stable lipid bilayer formation, (a). Illustration of the double-well (DW) chip used for ion channel measurement with the droplet contact method, (b). Schematic diagram of the channel current recordings for alpha-hemolysin reconstituted in the lipid bilayer, (c). Photograph of our portable system containing the DW chip with a handheld amplifier connected to a laptop PC, (d).

In this study, we propose a system for constructing a portable, low-noise, and reliable BLM experiments as a platform for conducting membrane protein measurements in outdoor environments. The stable and reproducible formation of BLM is achieved by a method referred to as the “droplet contact method” [8]–[13] in a double-well (DW) chip (Figure 1a,b). This chip has Ag/AgCl electrodes on the bottom of the chamber and is connected to a handheld amplifier via a laptop PC as shown in Figure 1b–d. We here apply this portable system for environmental nanopore sensing using alpha-hemolysin (αHL). [14] We also evaluate the applicability of this system for obtaining single channel current recordings in the field at high altitude (∼3623 m). This demonstration or experiment would be one of proof of concepts for our portable system in the outdoor operation.

## Material and Methods

### Materials

The following reagents were used in this study: poly(methyl methacrylate) (PMMA) substrate (Mitsubishi Rayon; Japan); KCl, K_2_HPO_4_, KH_2_PO_4_, and ethylenediamine tetraacetic acid (EDTA; Wako; Japan); 1,2-diphytanoyl-*sn*-glycero-3-phosphocholine (DPhPC) and phosphocholine from egg yolk (EggPC) (Avanti Polar Lipids; Alabama); *n*-decane (Sigma-Aldrich; St. Louis). Buffered electrolyte solutions were prepared from ultrapure water. The ultrapure water was prepared with >18 MΩ cm water from a Milli-Q system (Millipore). Wild-type αHL (Sigma-Aldrich; St. Louis) was obtained as a monomer polypeptide isolated from *Staphylococcus aureus* in the form of a lyophilized powder and dissolved at a concentration of 1.0 mg protein/mL in ultrapure water. During use, samples were diluted to the desired concentration using a buffered electrolyte solution, and stored at 4°C. For measurements in the field, all samples were stored at room temperature.

### Device fabrication

The device consists of a DW chip with the wells separated by a thin poly(chloro-*p*-xylylene) (parylene) film with micropores (described below). [13] The DW chip was made from poly(menthyl methacrylate) with dimensions of 30×20×4 mm, and fabricated using an automated CAD/CAM (computer aided design/ computer aided manufacturing) modeling machine (MM-100; Modia Systems; Japan). Each well was 4 mm in diameter and 3 mm in depth. The intersectional plane of the overlapped area of the wells in which the parylene film was settled was 2 mm in width.

The parylene film was fabricated using a general photolithography method. First, a 5-µm-thick parylene film was coated on a single-crystalline silicon substrate with chemical vapor deposition. Then, a thin aluminum layer was deposited on the parylene film and patterned using a standard photolithographic process. Using the aluminum layer as a mask, the exposed parylene film was etched by oxygen plasma. After the aluminum layer was removed, the parylene sheet with micropores (5 pores 100 µm or 150 µm in diameter) was peeled off of the silicon substrate using tweezers.

Ag/Cr was deposited and patterned on the PMMA substrate as wired electrodes for the electrical recording from the chambers to a handheld patch-clamp amplifier. The chambers with the parylene films and the wired substrate were connected using thermocompression bonding. The bottoms of the chambers, which made contact with droplets, were coated with Ag/AgCl paste (BAS; Japan).

### Lipid bilayer preparation and channel reconstitution using the droplet contact method

The droplet contact method for BLM formation is relatively simple comparing to conventional method such as painting method [15]. In addition, the BLM formed in our system showed around 2 weeks stable life time with reconstituting alamethicin channels. [11] First, the DPhPC or EggPC/*n*-decane (20 mg/mL) solution (5–7 µL) was initially dropped in each well. Next, the buffer solution (18–20 µL) was dropped into both wells. Within a few minutes of adding the buffer solution, the lipid monolayers made contact, and the BLM was formed. If the BLM becomes ruptured during the process, it can be reformed by repainting using a hydrophobic stick (Plastic needle, As one, Japan).

We defined the two droplets set on the working and ground electrodes as droplet A and B, respectively. A symmetrical buffer solution (1 M KCl, 10 mM phosphate-buffered saline (PBS), 1 mM EDTA, pH 7.4) was used for both droplets in this study. αHL was dissolved in droplet at a 0.6 µM concentration.

### Channel measurements

The channel currents were monitored using Pico or Pico2 (Tecella; CA) handheld amplifier and Axopatch 200B (Axon Instruments) patch-clamp amplifiers with a Digidata 1440A digitizer (Molecular Devices). The signals were detected through a 5-kHz low-pass filter at a sampling frequency of 20 kHz in Pico (and Pico2) or a 1-kHz low-pass filter at a sampling frequency of 5 kHz in Axopatch 200B at 23±1°C. Analysis of the channel current was performed using pCLAMP ver. 10.6 (Molecular Devices; Sunnyvale) and Igor Pro 6.2 (Wavemetrics; Oregon).

### Noise validation of the channel current in the system

The noise validation was carried out using two different system types: one in which the Ag/AgCl electrodes were directly inserted into the aqueous droplets using an electrode holder (PC-3; Nihonkoden; Japan), and another in which the electrodes were wired onto the bottom of the wells as described above. Axopatch 200B was used for the current measurement with a 1-kHz low-pass filter at a sampling frequency of 5 kHz and RMS (root mean square) noise. The headstage (RMS) noise between Axopatch 200B (0.5 pA) and PICO2 (0.3 pA) is similar in laboratory. The power spectrum was measured from the mean current noise of the single αHL channel (reconstituted in DPhPC) signal in 1 M KCl PBS buffer solution at 23±1°C.

### Field testing

The field test of the portable system was performed at the summit of Mount Fuji in Japan using our DW chip connected to a handheld amplifier and a laptop PC (Toshiba Dynabook R730; Japan) on August 5–6, 2011. The weight of this portable system is relatively right (less than 3 kg) compared with the conventional lipid bilayer system. The altitude, wind speed, and barometric pressure at the time of testing were measured using ADC Pro (Brunton; USA). All chemicals and equipment were transported up the mountain by four people (R. K., K. K., Y. T., and T. K.).

## Results and Discussion

### Reducing current noise by wiring electrodes on the bottom of the well

**Figure 2:**
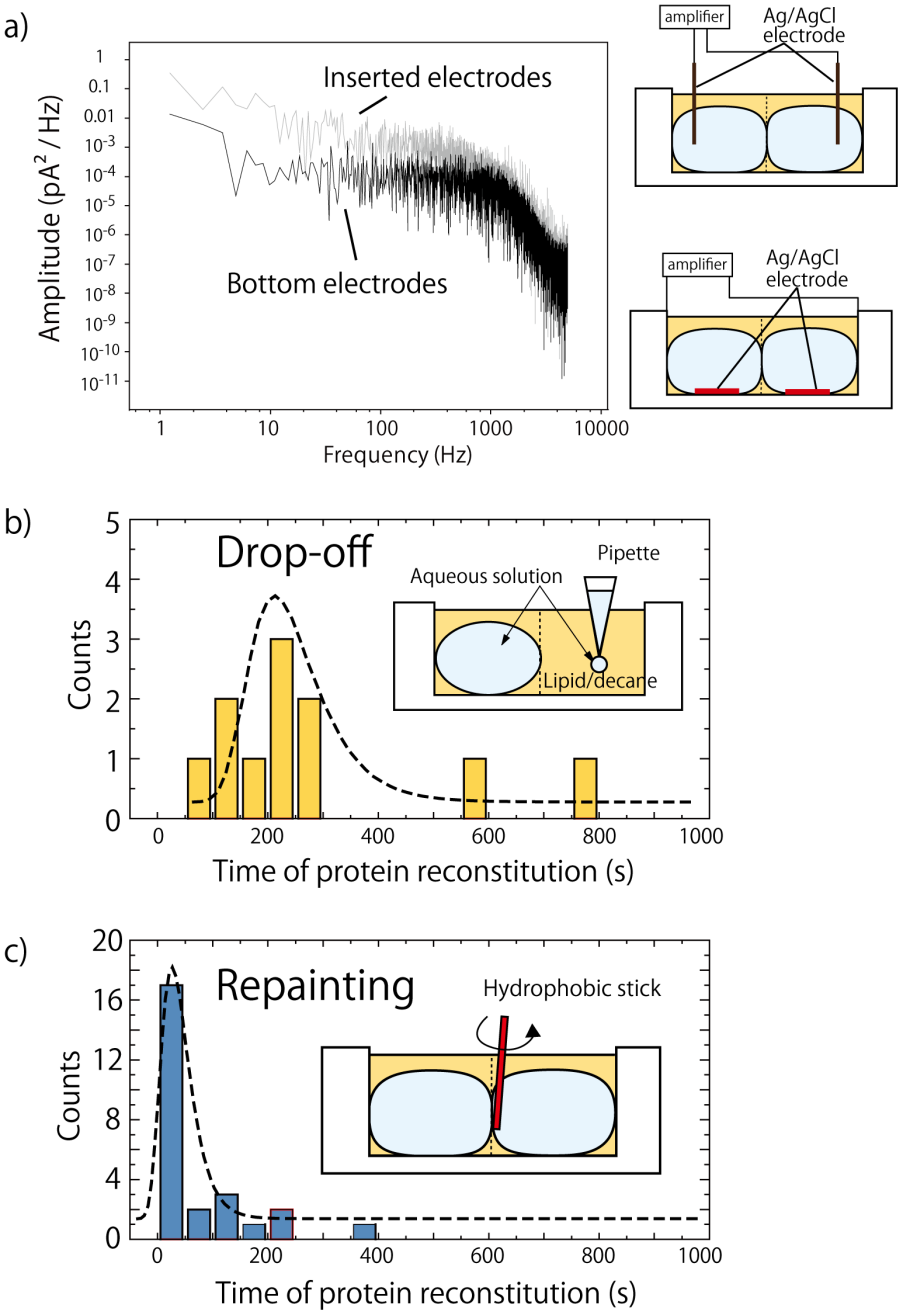
Frequency-dependent power density and diagrams of two different electrode systems: inserted and bottom-well, (a). Time required for αHL reconstitution using the drop-off (b) and repainting (c) methods, (b,c).

In a conventional system, the Ag/AgCl electrodes for recording ion channel currents are often directly inserted into the buffer solution (see the schematic in Figure 2a). While this is an efficient method for laboratory-based experiments, this potentially faltering configuration is not applicable to a hand-held system. Bottom-well wiring is a common technique used in MEMS (Micro-Electro-Mechanical Systems) fabrications; thus, we attempted to apply such wiring to our device and examined the current noise, which can be influenced by the electrode configuration. Figure 2a shows the power spectra of the αHL channel current noise from the two different systems (electrodes inserted into the aqueous droplets or positioned on the bottom of wells). The frequency-dependent noise characteristics were improved in the bottom-electrode system. The power density on the wiring system was over 1 order of magnitude lower for frequencies below 1000 Hz, and also showed significant reduction at low frequencies. For the inserted electrode system, the Ag/AgCl electrodes have several interfaces, aqueous buffer/*n*-decane and *n*-decane/air. Capacitor noise should thus be generated from these interfaces. [16] On the other hand, the electrodes in the bottom-electrode system only contact the aqueous phase without interfaces. The aqueous solution will be below the oil phase, and can be wetted over the electrode surface because the density of aqueous solution is greater than that of *n*-decane. [9] In addition, RMS noise (baseline noise) in the inserted and the bottom-electrode systems at 1 kHz filtering was determined to be 2.4±0.4 pA and 0.9±0.2 pA, respectively (the headstage noise of our equipment was approximately 0.5 pA RMS at the same conditions). These results demonstrated the advantage of the bottom-electrode system for obtaining low-noise channel recordings.

### Rapid αHL channel current measurement

The mechanism of αHL channel formation involves initial formation of the BLM by contact between the two lipid monolayers, and then the subsequent insertion of the αHL monomers in the BLM, which are then assembled and ultimately form a nanopore. Despite the versatility of this approach for BLM formation and channel reconstitution, the time of the channel formation are not clear. The time required for the appearance of an initial αHL current signal was examined using two different methods: drop-off or repainting method (see Materials and Methods). Figure 2b presents the histogram representing the time of initial αHL reconstitution using the drop-off method. The most frequent time required for the reconstitution was approximately 4 min after dropping the αHL solution. However, the time required when using the repainting method was approximately 10 times faster than that for the drop-off method (Figure 2c). The lipid phase can be deformed and the lipid bilayer is readily reformed by simply stroking the contact area using a hydrophobic stick. This method is highly useful for repeated measurements and supports the rapid data acquisition ability of this method.

### Field test: channel current measurement of αHL at high altitude

**Figure 3:**
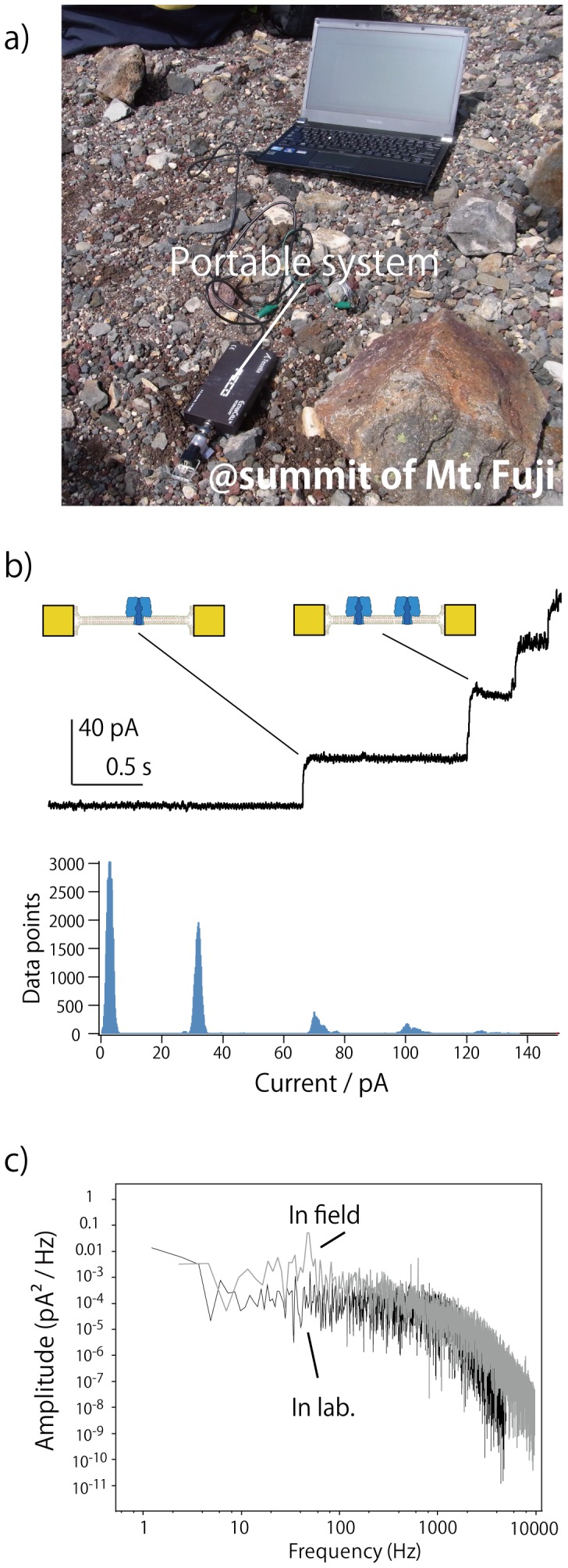
Photograph of the recording system, DW chip with an amplifier connected to a PC, at a high-altitude site (near the summit of Mount Fuji), (a). Current time frequency histogram of actual data taken at the field site, (b). Power density spectra of the open channel current of αHL in the field and the laboratory, (c).

The portability of a BLM system for nanopore or membrane receptor sensing is one of the essential requirements of obtaining environmental measurements in a natural area. To exhibit this ability using our chip and a handheld amplifier, we evaluated the single-channel measurement of αHL at a high-altitude location (the summit of Mount Fuji in Japan). The experimental setting is depicted in Figure 3a. The actual altitude was 3623 m. The DW chip with the handheld amplifier connected to a laptop personal computer (PC) via a USB (Universal Serial Bus) cable was placed on the ground, and the bilayer formation procedure was conducted. We found that the current noise of αHL was relatively low even without grounding in the natural environment, as shown in Figure 3b; the low noise current is likely due to the lack of electrical noise sources in the area. Nonetheless, we sometimes observed vibration noises that was generated by strong winds. The channel conductance of αHL was ∼1 nS, which is similar to the value measured in the laboratory. The frequency-dependence data of the current noise measured in the laboratory and in the field are shown in Figure 3c. The power density of the field measurement was over 1 order of magnitude higher than that of the laboratory measurement below 100 Hz, which can be caused by the vibration noises. In addition, at 50 Hz, electromagnetic noise clearly appeared in the field measurement, which was likely generated from the laptop PC.

In summary, a BLM was prepared in a DW chip connected to a handheld amplifier and a PC. The portable chip with bottom electrodes enabled low-electrical noise measurement. Because of the reproducible and stable BLM formation in the chip, the channel current signal of αHL could be measured at the single-molecule level at high altitude in a natural environment. We believe that this portable system would be applied to a wide variety of environmental measurements including biomimetic separation (e.g. nano-filtration of water [17]), measurement for deleterious gases [14] and chemical agent of odors in nature [18].
